# 9-(Biphenyl-4-yl­oxycarbon­yl)-10-methyl­acridinium trifluoro­methane­sulfonate

**DOI:** 10.1107/S1600536809007569

**Published:** 2009-03-14

**Authors:** Damian Trzybiński, Magdalena Skupień, Karol Krzymiński, Artur Sikorski, Jerzy Błażejowski

**Affiliations:** a Faculty of Chemistry, University of Gdańsk, J. Sobieskiego 18, 80-952 Gdańsk, Poland

## Abstract

In the crystal structure of the title compound, C_27_H_20_NO_2_^+^·CF_3_SO_3_^−^, the cations form inversion dimers through π–π inter­actions between the acridine ring systems [centroid-centroid distances = 3.668 (2)–3.994 (2) Å]. These dimers are further linked by C—H⋯O and C—H⋯π inter­actions. The cation and the anion are connected by C—H⋯O inter­actions. The mean plane of the acridine ring system makes dihedral angles of 10.6 (1) and 82.5 (1)°, respectively, with the adjacent phenyl ring and the carb­oxy group. The two phenyl rings of the biphenyl group are oriented at 42.9 (1)°.

## Related literature

For general background, see: Adamczyk *et al.* (2004 ▶); Becker *et al.* (1999 ▶); Dodeigne *et al.* (2000 ▶); Rak *et al.* (1999 ▶); Zomer & Jacquemijns (2001 ▶). For related structures, see: Sikorski *et al.* (2007 ▶, 2008 ▶). For mol­ecular inter­actions, see: Bianchi *et al.* (2004 ▶); Hunter & Sanders (1990 ▶); Steiner (1999 ▶); Takahashi *et al.* (2001 ▶). For the synthesis, see: Sato (1996 ▶); Sikorski *et al.* (2007 ▶).
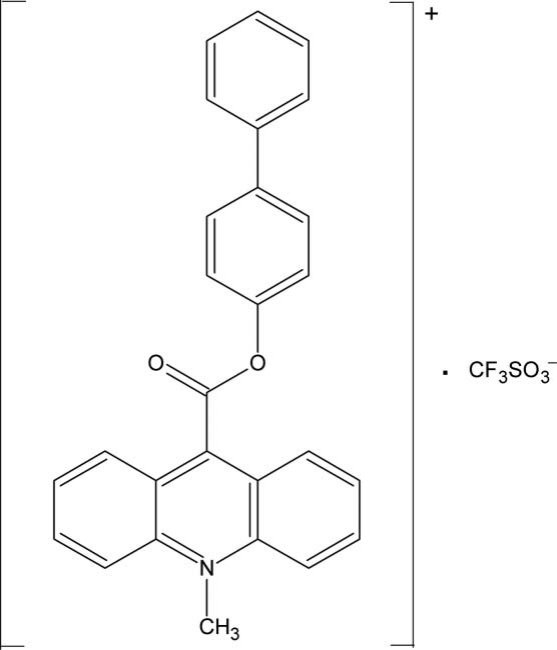


## Experimental

### 

#### Crystal data

C_27_H_20_NO_2_^+^·CF_3_SO_3_^−^
*M* *_r_* = 539.52
Monoclinic, 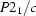

*a* = 9.4619 (2) Å
*b* = 12.4558 (5) Å
*c* = 20.7903 (7) Å
β = 94.559 (3)°
*V* = 2442.50 (14) Å^3^
*Z* = 4
Mo *K*α radiation
μ = 0.20 mm^−1^
*T* = 295 K
0.6 × 0.12 × 0.1 mm

#### Data collection

Oxford Diffraction GEMINI R ULTRA Ruby CCD diffractometer
Absorption correction: multi-scan (**CrysAlis RED**; Oxford Diffraction, 2008 ▶) *T*_min_ = 0.887, *T*_max_ = 0.977
42490 measured reflections
4408 independent reflections
3454 reflections with *I* > 2σ(*I*)
*R*_int_ = 0.033

#### Refinement

*R*[*F* ^2^ > 2σ(*F* ^2^)] = 0.041
*wR*(*F* ^2^) = 0.112
*S* = 1.05
4408 reflections
344 parameters
H-atom parameters constrained
Δρ_max_ = 0.22 e Å^−3^
Δρ_min_ = −0.39 e Å^−3^

### 

Data collection: *CrysAlis CCD* (Oxford Diffraction, 2008 ▶); cell refinement: *CrysAlis RED* (Oxford Diffraction, 2008 ▶); data reduction: *CrysAlis RED*; program(s) used to solve structure: *SHELXS97* (Sheldrick, 2008 ▶); program(s) used to refine structure: *SHELXL97* (Sheldrick, 2008 ▶); molecular graphics: *ORTEP-3* (Farrugia, 1997 ▶); software used to prepare material for publication: *SHELXL97* and *PLATON* (Spek, 2009 ▶).

## Supplementary Material

Crystal structure: contains datablocks global, I. DOI: 10.1107/S1600536809007569/is2396sup1.cif

Structure factors: contains datablocks I. DOI: 10.1107/S1600536809007569/is2396Isup2.hkl

Additional supplementary materials:  crystallographic information; 3D view; checkCIF report

## Figures and Tables

**Table 1:** Hydrogen-bond geometry (Å, °)

*D*—H⋯*A*	*D*—H	H⋯*A*	*D*⋯*A*	*D*—H⋯*A*
C6—H6⋯O33^i^	0.93	2.58	3.228 (3)	127
C7—H7⋯O34	0.93	2.59	3.431 (3)	151
C8—H8⋯O32	0.93	2.52	3.335 (2)	147
C22—H22⋯O33^ii^	0.93	2.51	3.271 (3)	140
C28—H28⋯O34^iii^	0.93	2.52	3.429 (3)	166
C30—H30*A*⋯O17^i^	0.96	2.55	3.125 (2)	118
C29—H29⋯*Cg*2^iv^	0.93	2.81	3.417 (2)	123
C30—H30*A*⋯*Cg*4^i^	0.96	2.83	3.683 (2)	148

Symmetry codes: (i) 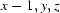; (ii) 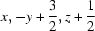; (iii) 
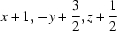
; (iv) 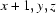. *Cg*2 and *Cg*4 are the centroids of the C1–C4/C11/C12 and C18–C23 rings, respectively.

**Table 2:** π–π Interactions (Å,°)

*I*	*J*	*CgI*⋯*CgJ*	Dihedral angle	*CgI*_Perp_	*CgJ*_Perp_	*CgI*_Offset_	*CgJ*_Offset_
1	1^v^	3.993 (2)		3.609 (2)	3.609 (2)	1.709 (2)	1.709 (2)
1	3^v^	3.668 (2)	2.0	3.583 (2)	3.578 (2)	0.785 (2)	0.807 (2)
2	3^v^	3.944 (2)	2.4	3.507 (2)	3.577 (2)	1.804 (2)	1.661 (2)

Symmetry codes: (v) 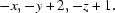

Notes: *Cg*1, *Cg*2 and *Cg*3 are the centroids of the C9/N10/C11–C14, C1–C4/C11/C12 and C5–C8/C13/C14  rings, respectively. *CgI*⋯*CgJ* is the distance between ring centroids. The dihedral angle is that between the planes of the rings *I* and *J*. *CgI*_Perp_ and *CgJ*_Perp_ are the perpendicular distances of *CgI* from ring *J* and of *CgJ* from ring *I*, respectively. *CgI*_Offset_ and *CgJ*_Offset_ are the distances between *CgI* and the perpendicular projection of *CgJ* on ring *I*, and between *CgJ* and the perpendicular projection of *CgI* on ring *J*, respectively.

**Fig. 1.**
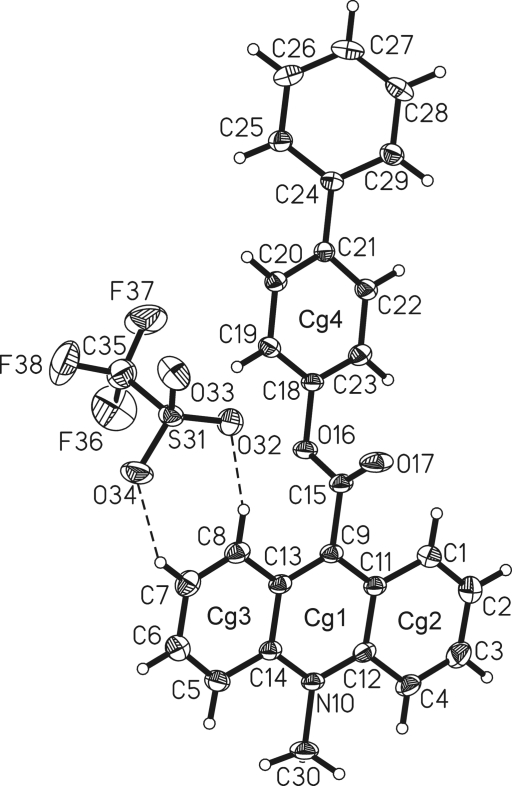
The molecular structure of the title compound, showing the atom-labeling scheme. Displacement ellipsoids are drawn at the 25% probability level, and H atoms are shown as small spheres of arbitrary radius. Cg1, Cg2, Cg3 and Cg4 denote the ring centroids.

**Fig. 2.**
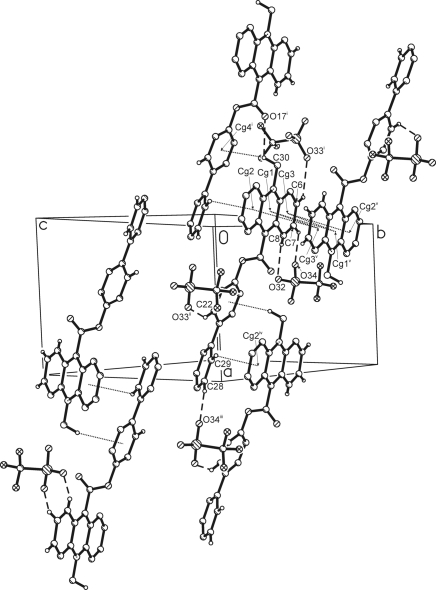
The arrangement of the ions in the crystal structure. The C–H···O interactions are represented by dashed lines, the C–H···π and π–π interactions by dotted lines. H atoms not involved in interactions have been omitted. [Symmetry codes: (i) x - 1, y, z; (ii) x, -y + 3/2, z + 1/2; (iii) x + 1, -y + 3/2, z + 1/2; (iv) x + 1, y, z; (v) -x, -y+2, -z + 1.]

## supplementary crystallographic information

### Comment

There has long been interest in phenyl 10-methylacridinium-9-carboxylates, owing to their distinctive chemiluminogenic features (Rak *et al.*, 1999; Dodeigne *et al.*, 2000; Zomer & Jacquemijns, 2001). Compounds of this kind are oxidized by hydrogen peroxide or other peroxides in alkaline media: the phenoxycarbonyl fragments are removed and the rest of the molecules are converted to electronically excited, light-emitting 10-methyl-9-acridinones. The above-mentioned chemiluminescence is the basis for using of phenyl 10-methylacridinium-9-carboxylates as chemiluminescent indicators, or as chemiluminogenic fragments of the chemiluminescent labels (Dodeigne *et al.*, 2000; Zomer & Jacquemijns, 2001) applied in assays of biologically and environmentally important entities such as antigens, antibodies, enzymes and DNA fragments (Becker *et al.*, 1999; Adamczyk *et al.*, 2004). As the structure of the phenyl fragment affects the efficiency of chemiluminescence (Zomer & Jacquemijns, 2001), we undertook investigations to enrich our knowledge of this effect. Here, we discuss the crystal structure of phenyl 10-methylacridinium-9-carboxylate substituted by phenyl in the phenyl fragment. The phenyl group, which enlarges the phenoxycarbonyl fragment removed during oxidation, may influence the stability and chemiluminescent efficiency of the compound investigated.

In the cation of the title compound (Fig. 1), the bond lengths and angles characterizing the geometry of the acridinium moiety are typical of acridine-based derivatives (Sikorski *et al.*, 2007, 2008). With respective average deviations from planarity of 0.026 (2) Å and 0.006 (2) Å, the acridine and benzene (C18—C23) ring systems in the cation are oriented at 10.6 (1)°. The C18—C23 and C24—C29 benzene ring systems, with respective average deviations from planarity of 0.006 (2) Å and 0.007 (2) Å, are mutually oriented at a dihedral angle of 42.9 (1)°. The carboxy group is twisted at an angle of 82.5 (1)° relative to the acridine skeleton. The mean planes of the acridine moieties are either parallel or are inclined at an angle of 21.1 (1)°.

In the crystal structure, the inversely oriented cations form dimers through multidirectional π–π interactions involving acridine moieties (Table 2, Fig. 2). These dimers are linked by C–H···O (Table 1, Fig. 2) and C–H···π (Table 1, Fig. 2) interactions to neighboring cations, and by C–H···O (Table 1, Fig. 2) interactions to neighboring anions. The C–H···O interactions are of the hydrogen-bond type (Steiner, 1999; Bianchi *et al.*, 2004). The C–H···π interactions should be of an attractive nature (Takahashi *et al.*, 2001), like the π–π interactions (Hunter & Sanders, 1990). The crystal structure is stabilized by a network of these short-range specific interactions and by long-range electrostatic interactions between ions.

### Experimental

The compound was synthesized in three steps (Sikorski *et al.*, 2007). First, 9-(chlorocarbonyl)-acridine was produced by treating acridine-9-carboxylic acid with a tenfold molar excess of thionyl chloride. Then, esterification with biphenyl-4-ol was carried out in anhydrous dichloromethane in the presence of *N*,*N*-diethylethanamine and a catalytic amount of *N*,*N*-dimethyl-4-pyridinamine (room temperature, 15 h) (Sato, 1996). The crude product was purified chromatographically (SiO_2_, cyclohexane/ethyl acetate, 3/2 *v*/*v*). The biphenyl-4-yl acridine-9-carboxylate thus obtained was quaternalized with a fivefold molar excess of methyl trifluoromethanesulfonate dissolved in anhydrous dichloromethane. The crude 9-[(biphenyl-4-yloxy)carbonyl]-10-methylacridinium salt was dissolved in a small amount of ethanol, filtered and precipitated with a 25 *v*/*v* excess of diethyl ether (yield 50%). Yellow crystals suitable for X-ray investigations were grown from absolute ethanol solution (m.p. 241–243 K).

### Refinement

H atoms were positioned geometrically, with C—H = 0.93 and 0.96 Å for the aromatic and methyl H atoms, respectively, and constrained to ride on their parent atoms with *U*_iso_(H) = *xU*_eq_(C), where *x* = 1.2 for the aromatic and *x* = 1.5 for the methyl H atoms.

### Crystal data

**Table:**

C_27_H_20_NO_2_^+^·CF_3_SO_3_^−^	*F*(000) = 1112
*M**_r_* = 539.52	*D*_x_ = 1.467 Mg m^−^^3^
Monoclinic, *P*2_1_/*c*	Mo *K*α radiation, λ = 0.71073 Å
Hall symbol: -P 2ybc	Cell parameters from 1909 reflections
*a* = 9.4619 (2) Å	θ = 3.0–29.2°
*b* = 12.4558 (5) Å	µ = 0.20 mm^−^^1^
*c* = 20.7903 (7) Å	*T* = 295 K
β = 94.559 (3)°	Needle, yellow
*V* = 2442.50 (14) Å^3^	0.6 × 0.12 × 0.1 mm
*Z* = 4	

### Data collection

**Table:**

Oxford Diffraction GEMINI R ULTRA Ruby CCD diffractometer	4408 independent reflections
Radiation source: Enhance (Mo) X-ray Source	3454 reflections with *I* > 2σ(*I*)
graphite	*R*_int_ = 0.033
Detector resolution: 10.4002 pixels mm^-1^	θ_max_ = 25.3°, θ_min_ = 3.0°
ω scans	*h* = −11→11
Absorption correction: multi-scan (*CrysAlis RED*; Oxford Diffraction, 2008)	*k* = −14→14
*T*_min_ = 0.887, *T*_max_ = 0.977	*l* = −24→24
42490 measured reflections	

### Refinement

**Table:**

Refinement on *F*^2^	Primary atom site location: structure-invariant direct methods
Least-squares matrix: full	Secondary atom site location: difference Fourier map
*R*[*F*^2^ > 2σ(*F*^2^)] = 0.041	Hydrogen site location: inferred from neighbouring sites
*wR*(*F*^2^) = 0.112	H-atom parameters constrained
*S* = 1.05	*w* = 1/[σ^2^(*F*_o_^2^) + (0.0642*P*)^2^ + 0.3778*P*] where *P* = (*F*_o_^2^ + 2*F*_c_^2^)/3
4408 reflections	(Δ/σ)_max_ = 0.001
344 parameters	Δρ_max_ = 0.22 e Å^−^^3^
0 restraints	Δρ_min_ = −0.39 e Å^−^^3^

### Special details

**Table:**

Geometry. All e.s.d.'s (except the e.s.d. in the dihedral angle between two l.s. planes) are estimated using the full covariance matrix. The cell e.s.d.'s are taken into account individually in the estimation of e.s.d.'s in distances, angles and torsion angles; correlations between e.s.d.'s in cell parameters are only used when they are defined by crystal symmetry. An approximate (isotropic) treatment of cell e.s.d.'s is used for estimating e.s.d.'s involving l.s. planes.

### Fractional atomic coordinates and isotropic or equivalent isotropic displacement parameters (Å^2^)

**Table:**

	*x*	*y*	*z*	*U*_iso_*/*U*_eq_	
C1	0.0826 (2)	0.85546 (17)	0.66803 (10)	0.0559 (5)	
H1	0.1794	0.8419	0.6743	0.067*	
C2	0.0070 (2)	0.8721 (2)	0.71966 (11)	0.0666 (6)	
H2	0.0516	0.8705	0.7612	0.080*	
C3	−0.1399 (2)	0.8920 (2)	0.71009 (11)	0.0661 (6)	
H3	−0.1913	0.9025	0.7459	0.079*	
C4	−0.2082 (2)	0.89610 (17)	0.65079 (10)	0.0556 (5)	
H4	−0.3052	0.9097	0.6462	0.067*	
C5	−0.1940 (2)	0.87413 (17)	0.41735 (10)	0.0533 (5)	
H5	−0.2913	0.8854	0.4114	0.064*	
C6	−0.1171 (2)	0.86205 (18)	0.36536 (11)	0.0608 (6)	
H6	−0.1632	0.8652	0.3242	0.073*	
C7	0.0303 (2)	0.84498 (17)	0.37199 (11)	0.0589 (5)	
H7	0.0807	0.8385	0.3356	0.071*	
C8	0.0983 (2)	0.83809 (16)	0.43131 (10)	0.0506 (5)	
H8	0.1956	0.8261	0.4355	0.061*	
C9	0.08977 (17)	0.84149 (13)	0.54978 (9)	0.0411 (4)	
N10	−0.19839 (14)	0.88388 (11)	0.53452 (7)	0.0409 (4)	
C11	0.01625 (17)	0.85832 (14)	0.60431 (9)	0.0426 (4)	
C12	−0.13276 (18)	0.87986 (14)	0.59538 (9)	0.0432 (4)	
C13	0.02305 (17)	0.84892 (13)	0.48766 (9)	0.0407 (4)	
C14	−0.12638 (17)	0.86960 (13)	0.48029 (9)	0.0405 (4)	
C15	0.24609 (17)	0.81633 (15)	0.55824 (9)	0.0442 (4)	
O16	0.26663 (11)	0.71126 (10)	0.55470 (6)	0.0468 (3)	
O17	0.33626 (14)	0.88239 (12)	0.56714 (9)	0.0750 (5)	
C18	0.40924 (17)	0.67406 (14)	0.56630 (9)	0.0397 (4)	
C19	0.48541 (18)	0.65153 (15)	0.51460 (9)	0.0453 (4)	
H19	0.4465	0.6623	0.4726	0.054*	
C20	0.62226 (18)	0.61225 (15)	0.52661 (8)	0.0443 (4)	
H20	0.6754	0.5962	0.4921	0.053*	
C21	0.68130 (17)	0.59638 (14)	0.58923 (8)	0.0378 (4)	
C22	0.59895 (19)	0.61933 (16)	0.63985 (9)	0.0472 (4)	
H22	0.6366	0.6088	0.6821	0.057*	
C23	0.46215 (19)	0.65756 (16)	0.62865 (9)	0.0493 (5)	
H23	0.4072	0.6718	0.6628	0.059*	
C24	0.82954 (17)	0.55657 (14)	0.60215 (8)	0.0385 (4)	
C25	0.88247 (19)	0.47452 (16)	0.56618 (9)	0.0497 (5)	
H25	0.8243	0.4422	0.5336	0.060*	
C26	1.0212 (2)	0.44002 (18)	0.57816 (11)	0.0587 (5)	
H26	1.0549	0.3842	0.5539	0.070*	
C27	1.1092 (2)	0.48737 (19)	0.62543 (11)	0.0603 (6)	
H27	1.2029	0.4649	0.6327	0.072*	
C28	1.0580 (2)	0.56810 (19)	0.66193 (11)	0.0607 (6)	
H28	1.1171	0.6003	0.6943	0.073*	
C29	0.91906 (19)	0.60188 (16)	0.65086 (9)	0.0505 (5)	
H29	0.8850	0.6558	0.6765	0.061*	
C30	−0.35306 (18)	0.90607 (18)	0.52817 (11)	0.0586 (5)	
H30A	−0.4013	0.8538	0.5524	0.088*	
H30B	−0.3875	0.9021	0.4835	0.088*	
H30C	−0.3703	0.9766	0.5445	0.088*	
S31	0.43886 (5)	0.83310 (5)	0.31913 (2)	0.05338 (17)	
O32	0.42879 (16)	0.85116 (15)	0.38681 (7)	0.0738 (5)	
O33	0.56626 (19)	0.87108 (17)	0.29520 (8)	0.0896 (6)	
O34	0.31337 (17)	0.85327 (16)	0.27869 (8)	0.0865 (6)	
C35	0.4565 (3)	0.6904 (2)	0.31430 (13)	0.0827 (8)	
F36	0.3429 (3)	0.64219 (17)	0.33492 (13)	0.1497 (9)	
F37	0.5652 (2)	0.65377 (17)	0.35025 (10)	0.1352 (8)	
F38	0.4689 (3)	0.65853 (16)	0.25421 (10)	0.1447 (9)	

### Atomic displacement parameters (Å^2^)

**Table:**

	*U*^11^	*U*^22^	*U*^33^	*U*^12^	*U*^13^	*U*^23^
C1	0.0429 (10)	0.0619 (13)	0.0621 (13)	0.0029 (9)	−0.0010 (9)	−0.0086 (10)
C2	0.0657 (14)	0.0780 (16)	0.0557 (13)	0.0022 (12)	0.0015 (11)	−0.0135 (11)
C3	0.0630 (13)	0.0762 (16)	0.0614 (14)	0.0035 (11)	0.0188 (11)	−0.0153 (11)
C4	0.0403 (10)	0.0604 (13)	0.0681 (14)	0.0041 (9)	0.0168 (10)	−0.0122 (10)
C5	0.0399 (10)	0.0544 (12)	0.0645 (13)	0.0030 (9)	−0.0023 (9)	−0.0015 (10)
C6	0.0603 (13)	0.0672 (15)	0.0542 (12)	0.0026 (11)	−0.0006 (10)	−0.0018 (10)
C7	0.0614 (13)	0.0612 (13)	0.0559 (13)	−0.0003 (10)	0.0170 (10)	−0.0024 (10)
C8	0.0394 (9)	0.0508 (12)	0.0631 (13)	0.0009 (8)	0.0135 (9)	−0.0019 (9)
C9	0.0294 (8)	0.0343 (10)	0.0597 (11)	−0.0011 (7)	0.0052 (8)	−0.0027 (8)
N10	0.0263 (7)	0.0368 (8)	0.0598 (10)	0.0017 (6)	0.0048 (6)	−0.0044 (7)
C11	0.0323 (8)	0.0389 (10)	0.0568 (11)	0.0007 (7)	0.0045 (8)	−0.0047 (8)
C12	0.0357 (9)	0.0346 (10)	0.0600 (12)	−0.0001 (7)	0.0095 (8)	−0.0063 (8)
C13	0.0317 (8)	0.0333 (9)	0.0577 (11)	−0.0008 (7)	0.0079 (8)	−0.0023 (8)
C14	0.0328 (8)	0.0312 (9)	0.0575 (11)	−0.0004 (7)	0.0048 (8)	−0.0028 (7)
C15	0.0292 (9)	0.0450 (11)	0.0588 (12)	0.0014 (8)	0.0070 (8)	−0.0039 (8)
O16	0.0289 (6)	0.0435 (7)	0.0673 (8)	0.0036 (5)	−0.0001 (5)	−0.0044 (6)
O17	0.0305 (7)	0.0491 (9)	0.1453 (16)	−0.0025 (6)	0.0065 (8)	−0.0091 (9)
C18	0.0279 (8)	0.0392 (10)	0.0518 (11)	0.0032 (7)	0.0021 (7)	−0.0023 (7)
C19	0.0388 (9)	0.0560 (12)	0.0401 (10)	0.0052 (8)	−0.0025 (8)	0.0003 (8)
C20	0.0368 (9)	0.0562 (12)	0.0403 (10)	0.0056 (8)	0.0063 (8)	−0.0029 (8)
C21	0.0356 (9)	0.0363 (9)	0.0413 (9)	0.0008 (7)	0.0020 (7)	−0.0009 (7)
C22	0.0426 (10)	0.0601 (12)	0.0383 (10)	0.0084 (9)	−0.0003 (8)	0.0004 (8)
C23	0.0426 (10)	0.0609 (12)	0.0456 (11)	0.0071 (9)	0.0108 (8)	−0.0043 (9)
C24	0.0335 (8)	0.0389 (10)	0.0429 (10)	0.0022 (7)	0.0016 (7)	0.0068 (7)
C25	0.0396 (9)	0.0508 (12)	0.0589 (12)	0.0032 (8)	0.0044 (8)	−0.0043 (9)
C26	0.0483 (11)	0.0570 (13)	0.0730 (14)	0.0149 (10)	0.0175 (10)	0.0068 (10)
C27	0.0351 (10)	0.0697 (15)	0.0759 (14)	0.0100 (10)	0.0028 (10)	0.0266 (12)
C28	0.0449 (11)	0.0656 (14)	0.0683 (14)	−0.0025 (10)	−0.0171 (10)	0.0106 (11)
C29	0.0460 (10)	0.0477 (11)	0.0562 (12)	0.0035 (9)	−0.0062 (9)	0.0011 (9)
C30	0.0273 (9)	0.0701 (14)	0.0787 (15)	0.0081 (9)	0.0060 (9)	−0.0022 (11)
S31	0.0446 (3)	0.0750 (4)	0.0403 (3)	0.0105 (2)	0.0021 (2)	0.0059 (2)
O32	0.0688 (10)	0.1071 (13)	0.0460 (9)	0.0136 (9)	0.0071 (7)	−0.0074 (8)
O33	0.0748 (11)	0.1331 (16)	0.0616 (10)	−0.0285 (11)	0.0099 (8)	0.0162 (10)
O34	0.0632 (10)	0.1304 (16)	0.0636 (10)	0.0386 (10)	−0.0095 (8)	0.0109 (10)
C35	0.094 (2)	0.0887 (19)	0.0651 (16)	0.0174 (16)	0.0063 (14)	0.0031 (14)
F36	0.160 (2)	0.1052 (16)	0.185 (2)	−0.0438 (14)	0.0162 (17)	0.0355 (14)
F37	0.1460 (17)	0.1334 (16)	0.1238 (16)	0.0835 (14)	−0.0050 (13)	0.0290 (12)
F38	0.231 (3)	0.1055 (14)	0.0970 (14)	0.0430 (15)	0.0084 (15)	−0.0326 (11)

### Geometric parameters (Å, °)

**Table:**

C1—C2	1.352 (3)	C19—C20	1.388 (2)
C1—C11	1.421 (3)	C19—H19	0.9300
C1—H1	0.9300	C20—C21	1.389 (2)
C2—C3	1.411 (3)	C20—H20	0.9300
C2—H2	0.9300	C21—C22	1.388 (2)
C3—C4	1.347 (3)	C21—C24	1.492 (2)
C3—H3	0.9300	C22—C23	1.382 (3)
C4—C12	1.418 (3)	C22—H22	0.9300
C4—H4	0.9300	C23—H23	0.9300
C5—C6	1.358 (3)	C24—C25	1.384 (3)
C5—C14	1.411 (3)	C24—C29	1.388 (3)
C5—H5	0.9300	C25—C26	1.385 (3)
C6—C7	1.407 (3)	C25—H25	0.9300
C6—H6	0.9300	C26—C27	1.370 (3)
C7—C8	1.347 (3)	C26—H26	0.9300
C7—H7	0.9300	C27—C28	1.371 (3)
C8—C13	1.425 (3)	C27—H27	0.9300
C8—H8	0.9300	C28—C29	1.383 (3)
C9—C11	1.392 (3)	C28—H28	0.9300
C9—C13	1.395 (3)	C29—H29	0.9300
C9—C15	1.508 (2)	C30—H30A	0.9600
N10—C12	1.365 (2)	C30—H30B	0.9600
N10—C14	1.374 (2)	C30—H30C	0.9600
N10—C30	1.485 (2)	S31—O33	1.4212 (17)
C11—C12	1.433 (2)	S31—O34	1.4216 (16)
C13—C14	1.433 (2)	S31—O32	1.4356 (15)
C15—O17	1.189 (2)	S31—C35	1.788 (3)
C15—O16	1.326 (2)	C35—F37	1.305 (3)
O16—C18	1.4289 (19)	C35—F38	1.325 (3)
C18—C23	1.368 (3)	C35—F36	1.332 (3)
C18—C19	1.370 (3)		
			
C2—C1—C11	120.93 (19)	C18—C19—H19	120.9
C2—C1—H1	119.5	C20—C19—H19	120.9
C11—C1—H1	119.5	C19—C20—C21	121.24 (16)
C1—C2—C3	119.5 (2)	C19—C20—H20	119.4
C1—C2—H2	120.3	C21—C20—H20	119.4
C3—C2—H2	120.3	C22—C21—C20	118.23 (15)
C4—C3—C2	122.1 (2)	C22—C21—C24	120.56 (15)
C4—C3—H3	118.9	C20—C21—C24	121.21 (15)
C2—C3—H3	118.9	C23—C22—C21	121.20 (17)
C3—C4—C12	120.16 (18)	C23—C22—H22	119.4
C3—C4—H4	119.9	C21—C22—H22	119.4
C12—C4—H4	119.9	C18—C23—C22	118.63 (17)
C6—C5—C14	120.09 (18)	C18—C23—H23	120.7
C6—C5—H5	120.0	C22—C23—H23	120.7
C14—C5—H5	120.0	C25—C24—C29	117.95 (16)
C5—C6—C7	121.9 (2)	C25—C24—C21	121.59 (16)
C5—C6—H6	119.0	C29—C24—C21	120.46 (16)
C7—C6—H6	119.0	C24—C25—C26	120.71 (19)
C8—C7—C6	119.76 (19)	C24—C25—H25	119.6
C8—C7—H7	120.1	C26—C25—H25	119.6
C6—C7—H7	120.1	C27—C26—C25	120.6 (2)
C7—C8—C13	120.91 (18)	C27—C26—H26	119.7
C7—C8—H8	119.5	C25—C26—H26	119.7
C13—C8—H8	119.5	C26—C27—C28	119.42 (18)
C11—C9—C13	121.68 (16)	C26—C27—H27	120.3
C11—C9—C15	119.01 (17)	C28—C27—H27	120.3
C13—C9—C15	119.29 (16)	C27—C28—C29	120.3 (2)
C12—N10—C14	122.46 (14)	C27—C28—H28	119.9
C12—N10—C30	117.50 (15)	C29—C28—H28	119.9
C14—N10—C30	120.04 (16)	C28—C29—C24	121.01 (19)
C9—C11—C1	122.90 (17)	C28—C29—H29	119.5
C9—C11—C12	118.22 (17)	C24—C29—H29	119.5
C1—C11—C12	118.87 (17)	N10—C30—H30A	109.5
N10—C12—C4	121.77 (16)	N10—C30—H30B	109.5
N10—C12—C11	119.83 (15)	H30A—C30—H30B	109.5
C4—C12—C11	118.40 (18)	N10—C30—H30C	109.5
C9—C13—C8	122.43 (16)	H30A—C30—H30C	109.5
C9—C13—C14	118.75 (16)	H30B—C30—H30C	109.5
C8—C13—C14	118.82 (17)	O33—S31—O34	115.21 (11)
N10—C14—C5	122.53 (16)	O33—S31—O32	114.53 (10)
N10—C14—C13	118.99 (16)	O34—S31—O32	115.76 (10)
C5—C14—C13	118.47 (16)	O33—S31—C35	103.03 (14)
O17—C15—O16	125.81 (16)	O34—S31—C35	102.70 (13)
O17—C15—C9	123.99 (17)	O32—S31—C35	102.97 (12)
O16—C15—C9	110.20 (15)	F37—C35—F38	108.1 (2)
C15—O16—C18	116.83 (13)	F37—C35—F36	106.1 (2)
C23—C18—C19	122.49 (16)	F38—C35—F36	107.6 (3)
C23—C18—O16	118.58 (15)	F37—C35—S31	112.9 (2)
C19—C18—O16	118.86 (16)	F38—C35—S31	111.5 (2)
C18—C19—C20	118.19 (17)	F36—C35—S31	110.4 (2)
			
C11—C1—C2—C3	0.4 (3)	C13—C9—C15—O17	97.2 (2)
C1—C2—C3—C4	−0.7 (4)	C11—C9—C15—O16	97.79 (19)
C2—C3—C4—C12	0.3 (3)	C13—C9—C15—O16	−83.2 (2)
C14—C5—C6—C7	−0.1 (3)	O17—C15—O16—C18	3.8 (3)
C5—C6—C7—C8	1.4 (3)	C9—C15—O16—C18	−175.79 (14)
C6—C7—C8—C13	−0.6 (3)	C15—O16—C18—C23	84.4 (2)
C13—C9—C11—C1	−177.44 (17)	C15—O16—C18—C19	−98.6 (2)
C15—C9—C11—C1	1.6 (3)	C23—C18—C19—C20	−1.1 (3)
C13—C9—C11—C12	2.3 (3)	O16—C18—C19—C20	−178.00 (16)
C15—C9—C11—C12	−178.69 (16)	C18—C19—C20—C21	−0.2 (3)
C2—C1—C11—C9	−179.9 (2)	C19—C20—C21—C22	1.0 (3)
C2—C1—C11—C12	0.4 (3)	C19—C20—C21—C24	−178.56 (17)
C14—N10—C12—C4	179.19 (17)	C20—C21—C22—C23	−0.4 (3)
C30—N10—C12—C4	0.0 (2)	C24—C21—C22—C23	179.15 (18)
C14—N10—C12—C11	−1.1 (2)	C19—C18—C23—C22	1.7 (3)
C30—N10—C12—C11	179.76 (16)	O16—C18—C23—C22	178.58 (17)
C3—C4—C12—N10	−179.76 (19)	C21—C22—C23—C18	−0.9 (3)
C3—C4—C12—C11	0.5 (3)	C22—C21—C24—C25	137.96 (19)
C9—C11—C12—N10	−0.3 (2)	C20—C21—C24—C25	−42.5 (3)
C1—C11—C12—N10	179.44 (16)	C22—C21—C24—C29	−42.4 (3)
C9—C11—C12—C4	179.45 (17)	C20—C21—C24—C29	137.10 (19)
C1—C11—C12—C4	−0.8 (3)	C29—C24—C25—C26	−0.8 (3)
C11—C9—C13—C8	176.29 (17)	C21—C24—C25—C26	178.82 (17)
C15—C9—C13—C8	−2.7 (3)	C24—C25—C26—C27	−0.7 (3)
C11—C9—C13—C14	−2.9 (2)	C25—C26—C27—C28	1.4 (3)
C15—C9—C13—C14	178.10 (15)	C26—C27—C28—C29	−0.4 (3)
C7—C8—C13—C9	179.55 (18)	C27—C28—C29—C24	−1.2 (3)
C7—C8—C13—C14	−1.3 (3)	C25—C24—C29—C28	1.8 (3)
C12—N10—C14—C5	−179.76 (16)	C21—C24—C29—C28	−177.87 (17)
C30—N10—C14—C5	−0.6 (3)	O33—S31—C35—F37	61.9 (2)
C12—N10—C14—C13	0.5 (2)	O34—S31—C35—F37	−178.0 (2)
C30—N10—C14—C13	179.62 (16)	O32—S31—C35—F37	−57.4 (2)
C6—C5—C14—N10	178.39 (19)	O33—S31—C35—F38	−60.0 (2)
C6—C5—C14—C13	−1.8 (3)	O34—S31—C35—F38	60.1 (2)
C9—C13—C14—N10	1.5 (2)	O32—S31—C35—F38	−179.3 (2)
C8—C13—C14—N10	−177.72 (16)	O33—S31—C35—F36	−179.51 (19)
C9—C13—C14—C5	−178.30 (16)	O34—S31—C35—F36	−59.5 (2)
C8—C13—C14—C5	2.5 (2)	O32—S31—C35—F36	61.1 (2)
C11—C9—C15—O17	−81.8 (3)		

### Hydrogen-bond geometry (Å, °)

**Table:**

*D*—H···*A*	*D*—H	H···*A*	*D*···*A*	*D*—H···*A*
C6—H6···O33^i^	0.93	2.58	3.228 (3)	127
C7—H7···O34	0.93	2.59	3.431 (3)	151
C8—H8···O32	0.93	2.52	3.335 (2)	147
C22—H22···O33^ii^	0.93	2.51	3.271 (3)	140
C28—H28···O34^iii^	0.93	2.52	3.429 (3)	166
C30—H30A···O17^i^	0.96	2.55	3.125 (2)	118
C29—H29···Cg2^iv^	0.93	2.81	3.417 (2)	123
C30—H30A···Cg4^i^	0.96	2.83	3.683 (2)	148

Symmetry codes:  (i) *x*−1, *y*, *z*; (ii) *x*, −*y*+3/2, *z*+1/2; (iii) *x*+1, −*y*+3/2, *z*+1/2; (iv) *x*+1, *y*, *z*.

### Table 2 π–π Interactions (Å,°)

**Table:**

*I*	*J*	*CgI*···*CgJ*	Dihedral angle	*CgI*_Perp_	*CgJ*_Perp_	*CgI*_Offset_	*CgJ*_Offset_
1	1^v^	3.993 (2)	0	3.609 (2)	3.609 (2)	1.709 (2)	1.709 (2)
1	3^v^	3.668 (2)	2.0	3.583 (2)	3.578 (2)	0.785 (2)	0.807 (2)
2	3^v^	3.944 (2)	2.4	3.507 (2)	3.577 (2)	1.804 (2)	1.661 (2)

Symmetry codes: (v) -*x*, -*y*+2, -*z*+1.Notes: *Cg*1, *Cg*2 and *Cg*3 are the centroids of the C9/N10/C11–C14, C1–C4/C11/C12 and C5–C8/C13/C14 rings, respectively. *CgI*···*CgJ* is the distance between ring centroids. The dihedral angle is that between the planes of the rings *I* and *J*. *CgI*_Perp_ and *CgJ*_Perp_ are the perpendicular distances of *CgI* from ring *J* and of *CgJ* from ring *I*, respectively. *CgI*_Offset_ and *CgJ*_Offset_ are the distances between *CgI* and the perpendicular projection of *CgJ* on ring *I*, and between *CgJ* and the perpendicular projection of *CgI* on ring *J*, respectively.

## References

[bb1] Adamczyk, M., Fino, J. R., Mattingly, P. G., Moore, J. A. & Pan, Y. (2004). *Bioorg. Med. Chem. Lett.*, **14**, 2313–2317. 10.1016/j.bmcl.2004.01.105 15081031

[bb2] Becker, M., Lerum, V., Dickson, S., Nelson, N. C. & Matsuda, E. (1999). *Biochemistry*, **38**, 5601–5611. 10.1021/bi9828066 10220349

[bb3] Bianchi, R., Forni, A. & Pilati, T. (2004). *Acta Cryst.* **B**60, 559–568. 10.1107/S0108768104014557 15367791

[bb4] Dodeigne, C., Thunus, L. & Lejeune, R. (2000). *Talanta*, **51**, 415–439. 10.1016/s0039-9140(99)00294-5 18967873

[bb5] Farrugia, L. J. (1997). *J. Appl. Cryst.* **30**, 565.

[bb6] Hunter, C. A. & Sanders, J. K. M. (1990). *J. Am. Chem. Soc.* **112**, 5525–5534.

[bb7] Oxford Diffraction. (2008). *CrysAlis CCD* and *CrysAlis RED* Oxford Diffraction Ltd, Abingdon, England.

[bb8] Rak, J., Skurski, P. & Błażejowski, J. (1999). *J. Org. Chem.*, **64**, 3002–3008. 10.1021/jo980566u 11674394

[bb9] Sato, N. (1996). *Tetrahedron Lett.* **37**, 8519–8522.

[bb10] Sheldrick, G. M. (2008). *Acta Cryst.* **A**64, 112–122. 10.1107/S0108767307043930 18156677

[bb11] Sikorski, A., Krzymiński, K., Malecha, P., Lis, T. & Błażejowski, J. (2007). *Acta Cryst.* **E**63, o4484–o4485.

[bb12] Sikorski, A., Niziołek, A., Krzymiński, K., Lis, T. & Błażejowski, J. (2008). *Acta Cryst.* **E**64, o372–o373. 10.1107/S1600536807068109 PMC2960374 21201404

[bb13] Spek, A. L. (2009). *Acta Cryst.* **D**65, 148–155. 10.1107/S090744490804362X PMC2631630 19171970

[bb14] Steiner, T. (1999). *Chem. Commun.* pp. 313–314.

[bb15] Takahashi, O., Kohno, Y., Iwasaki, S., Saito, K., Iwaoka, M., Tomada, S., Umezawa, Y., Tsuboyama, S. & Nishio, M. (2001). *Bull. Chem. Soc. Jpn*, **74**, 2421–2430.

[bb16] Zomer, G. & Jacquemijns, M. (2001). *Chemiluminescence in Analytical Chemistry*, edited by A. M. Garcia-Campana & W. R. G. Baeyens, pp. 529–549. New York: Marcel Dekker.

